# Rheological Model to Describe the Cyclic Load-Bearing Behaviour of Strain-Hardening Cement-Based Composites (SHCC)

**DOI:** 10.3390/ma14216444

**Published:** 2021-10-27

**Authors:** Dominik Junger, Steffen Müller, Viktor Mechtcherine

**Affiliations:** Institute of Construction Materials, Technische Universität Dresden, 01187 Dresden, Germany; dominik.junger@tu-dresden.de (D.J.); viktor.mechtcherine@tu-dresden.de (V.M.)

**Keywords:** SHCC, strain-hardening, cyclic loading, damage mechanisms, rheological modelling

## Abstract

The mechanical behaviour of strain-hardening cement-based composites (SHCC) under monotonic tensile loading has been the subject of research for many years. The recent research on the SHCC’s performance under cyclic loading has enabled the identification of a wide variety of damage phenomena different to those observed under monotonic loading. The article at hand first summarises the experimental evidence of such phenomena in the context of the material performance observed. On this basis, the mechanisms behind these phenomena are discussed and explained using rheological modelling.

## 1. Introduction

**Figure 1:**
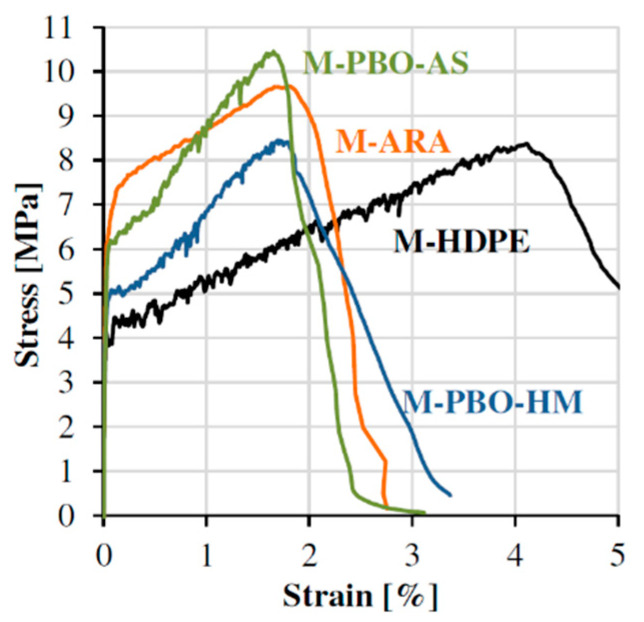
Mechanical behaviour of SHCC with different polymeric micro-fibres [4].

Strain-hardening cement-based composites (SHCC), also known as engineered cement-based composites (ECC) comprise a group of micro mechanic-based building materials demonstrating outstanding mechanical properties when subjected to tensile loading [1,2]. By adding specific high-performance microfibres, e.g., aramid fibres (ARA), polybenzoxazole fibres (as spun: PBO-AS; high modulus: PBO-HM) or high-density polyethylene fibres (HDPE), considerable enhancements on the order of several percent can be achieved in tensile strength and strain capacity [3,4]; see Figure 1.

The fine pattern of multiple cracks characteristic of SHCC ensures not only the high deformability and energy absorption in the structural member made of this material, but also a pronounced increase in its durability in comparison to conventional concrete structures [5]. First applications of SHCC in the practice of construction show a high potential of this novel material [6].

Most structures are subjected not only to monotonous and quasi-static loading, but also to cyclic loading-unloading processes. Hence, sound knowledge of the material behaviour under cyclic loading is critical to the safe design of load-bearing elements. In this, not only characteristic values need to be provided for a particular fibre-matrix combination, but the in-depth understanding of the damage processes is in its essence necessary. This would enable the adaptation of the material properties according to the expected loading in various design scenarios. Here, both numerical and rheological models can be used to describe the material’s behaviour and to predict its response after certain loading regimes. This even results in the possibility to evaluate the degradation, and therefore, the structural stability of a component after a serious load impact, e.g., earthquakes.

This article summarises the current state of knowledge on the damage mechanisms of SHCC under cyclic loading. After presenting the material composition and the test methods, the damage mechanisms found in respect of the individual loading conditions are discussed. Then, these damage mechanisms are described by a model composed of rheological elements. The complete model created in this way not only provides a physically based description of the materials’ behaviour, but also an understanding of the possible points of action with respect to purposeful material design and adaptation. A mathematical framework for the model is planned for publication in a follow-up article. The challenge here is to assign suitable parameters for individual elements or groups of elements, which are within the scope of the results of experimental investigations. These must then be statistically randomly applied to the overall model.

## 2. Materials and Methods

### 2.1. Material Composition

In examining the mechanical behaviour of SHCC under cyclic loading, a well-established composition previously designed at the TU Dresden was chosen, e.g., [7]; see Table 1. Apart from cement the mixture contains a high volume of fly ash as the binder material, which is instrumental in reducing the strong chemical bond of the hydrophilic polyvinylalcohol (PVA) fibres to the cement-based matrix, thus avoiding premature fibre rupture instead of beneficial, steady fibre delamination and eventual pull-out [4]. In addition to water, a superplasticizer and a viscosity agent were used to achieve the desired workability. The PVA fibres under investigation had a diameter of 40 µm and a length of 12 mm. Accordingly, very fine sand is employed as the aggregate to obtain uniform fibre distribution in the SHCC.

### 2.2. Testing Procedures

#### 2.2.1. Single Fibre Pull-Out

**Figure 2:**
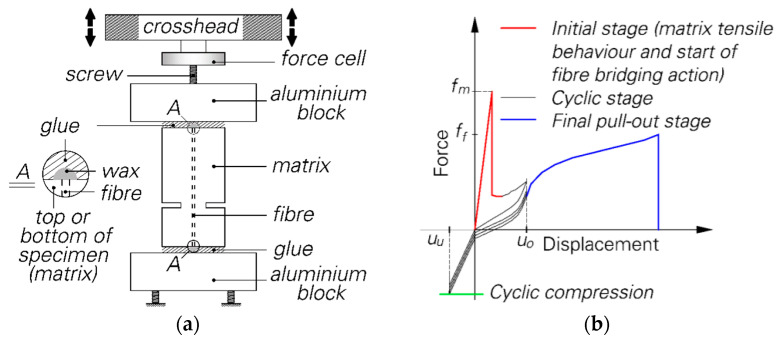
Double-sided, single fibre pull-out test: (**a**) testing setup [8]; (**b**) applied cyclic loading regime [9].

The crack-bridging behaviour of single PVA fibres under cyclic deformation-controlled loading was investigated using a novel double-sided pull-out testing setup developed by Ranjbarian and Mechtcherine [8]; see Figure 2a. The specimens for this test set-up were produced by fixing single fibres in a special mould filled with fresh matrix. Thin plastic foils were used to obtain the notches needed to force the crack to open exactly in the defined cross-section. The embedded lengths of the fibres were 2 mm and 6 mm. Detailed information about the casting procedure can be found in [8]. The loading regime for the pull-out tests at an age of 28 days is shown in Figure 2b; see [9] for details.

By applying this alternating loading regime in the cyclic stage and slightly distinct settings, the effects of various parameters on the pull-out response were investigated. In a preliminary study, Ranjbarian and Mechtcherine [10] studied the damage of PVA fibres subjected to cyclic loading. To examine the influence of loading parameters, Ranjbarian and Mechtcherine [9] pre-damaged the specimens at a loading rate of 0.01 mm/s until a crack-opening of 100 µm was reached. Afterwards cyclic loading was started with pre-defined upper and lower reversal points of 100 µm and 0.02, −0.01 or −0.025 mm, respectively, at a loading rate of 1 mm/s. After a certain number of loading cycles, the fibre was extracted. The same loading regime was applied to investigate the influence of initial crack width [11]. Here, a displacement increment per cycle was set to avoid reduced tensile forces with an increasing number of cycles. The initial crack widths were 40 and 100 µm. The tests were stopped when final crack-openings of 60 and 200 µm were reached. To determine the implication of fibre orientation on its mechanical behaviour, displacement-controlled pull-out tests with a displacement rate of 0.05 mm/s and upper and lower load-controlled reversal point of 0.5 and 0.2 N were carried out by Ranjbarian et al. [12] on single-sided, single fibre pull-out specimens. The defined fibre-inclination angles were 30° and 60°. Furthermore, Jun and Mechtcherine [13] investigated the cyclic tensile behaviour of PVA fibres in single-sided, single fibre pull-out tests at a displacement rate of 0.01 mm/s. In that work, the fibres were inserted into medical cannulas to achieve the proper embedded length and subsequently cast into the concrete matrix.

#### 2.2.2. Dumbbell-Shaped Specimens

**Figure 3:**
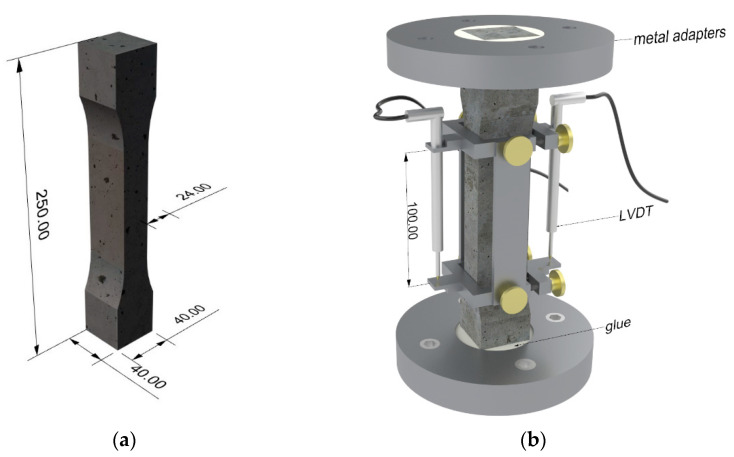
Macro-mechanical cyclic tests: (**a**) dumbbell-shaped specimen; (**b**) experimental setup.

In investigating the macro-mechanical behaviour of SHCC under cyclic loading, dumbbell-shaped specimens with a total length of 250 mm and dimensions of 40 mm × 24 mm × 100 mm in the measuring range were used; see Figure 3a. After mixing, the SHCC was cast in steel forms layer-by-layer to minimize voids in the specimens. Afterwards the forms were covered with plastic sheets and stored for two days. After de-moulding, the samples were stored for an additional 54 days under stable climate conditions, i.e., 20 °C, 65% RH. The cyclic tests were performed with non-rotatable boundary conditions. For this, a special fast-setting glue was used to fix the samples in metal adapters with conical openings, which were attached to a servo-hydraulic testing machine. Two linear variable differential transformers (LVDT) were installed to measure the deformations within a gauge length of 100 mm. The experimental setup is illustrated in Figure 3b.

Two different loading regimes explained hereinafter were applied to examine the mechanical behaviour of the composite.

##### Deformation-Controlled Regime

**Figure 4:**
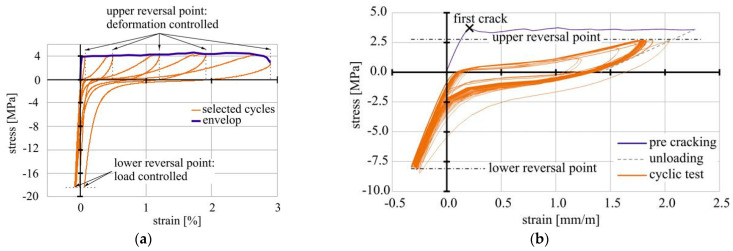
Schematic presentation of the testing regimes [7]: (**a**) deformation-controlled regime; (**b**) load-controlled regime.

Müller and Mechtcherine [6,7,14] performed pure cyclic tension tests such as alternating tension-compression tests on 56-day old, dumbbell-shaped specimens with deformation-controlled upper reversal points and load-controlled lower reversal points at a strain rate of 10^−2^·s^−1^. Distinct incremental deformations (0.2%, 0.002%, 0.0002%) were chosen for the tensile portion to pull the fibres out of the matrix gradually while the compressive reversal point was set to 0%, 25% and 50% of the compressive strength, respectively. The applied compression should induce complete crack closure and thus promote the fibres’ degradation through their increased crushing between the crack faces. The loading regime followed according to Figure 4a. Jun and Mechtcherine [13] also executed deformation-controlled, cyclic tensile tests at a loading rate of 0.01 mm/s and a deformation increment of 0.1 mm per cycle on dumbbell-shaped samples similar to that in Figure 3a as well as on larger specimens with a total length of 500 mm [15].

##### Load-Controlled Regime

Additionally, Müller and Mechtcherine performed load-controlled, cyclic uniaxial tension and tension-compression tests [7,16]. For this purpose, a loading regime was applied as shown in Figure 4b. Unlike the samples in the deformation-controlled tests, the specimens were pre-damaged until a deformation of 250 µm was reached before the cyclic loading with a frequency of 5 Hz was started. The lower reversal points were set to either 0.1 MPa in the case of pure cyclic tension tests or −8.0 MPa for alternating cyclic loading [16]. For the upper reversal point, the authors chose between two options. Either the individual first crack stress of each single specimen or an average first crack stress was defined as the reference level for the upper reversal point. On this basis, three different upper stress levels related to first crack stress were specified: 40%, 60% and 80%. In addition to deformation-controlled cyclic loading tests, Jun and Mechtcherine [15] also investigated the load-controlled uniaxial tension regime. After reaching a strain of 0.5% the specimens were cyclically loaded at a frequency of 0.5 Hz.

#### 2.2.3. Modelling Approaches

SHCCs make up a class of materials whose macroscopic composite properties are significantly influenced by meso- and microscopic failure mechanisms. On the material side, the processes can be assigned to the component’s fibre, cement-based matrix, and contact zone. To link the macroscopic composite behaviour and failure mechanisms on the meso- and microscale, material modelling should follow a multiscale approach that covers all essential experimentally determined phenomena. The first suggestion for such an approach was published by Jun and Mechtcherine [17]. Their analytical model is based on the experimental results of single fibre pull-out testing. The stress-crack opening relationship for the single crack is obtained by statistically accounting for the contributions of all fibres bridging the crack under consideration. The transition to the macroscopic model is created by serially linking the individual cracks and pieces of uncracked SHCC in between. The scatter of the material properties is explained using appropriate statistical functions.

An alternative, multiscale approach was proposed by Ranjbarian [18]. His model describes the bridging behaviour of a single crack at the composite level, taking the changing pull-out mechanisms due to the fibre inclination angle and the loading scenario into account. The experimental results were chiefly obtained using the newly developed single-fibre pull-out methods [8,9]. A major difference to previously available modelling approaches is the introduction of the non-simultaneity hypothesis. This proposes the determination of the bridging parameters based on the results of the bridging or pull-out behaviour of all samples in a series. In conventional approaches, the parameters are determined for each sample individually and then used as an average value, independent of the crack-opening movement. Comparison calculations with results of other fibre and load types from Curosu et al. [4,19] showed good agreement between the experimental and numerical data.

As an alternative to the approaches mentioned, material behaviour can also be described using the finite element method. The particular challenge here is the formulation of suitable elements which can represent the large strains and displacements that occur during fibre pull-out. Detailed information on this can be found in the numerical considerations of Storm et al. [20,21,22] based on the experimental work of Ranjbarian [8,9,12].

The modelling approaches presented in the previous sections describe the material behaviour of highly ductile concrete and similar materials in a very high level of detail. This is all too complex and extensive in its adjustment possibilities for the user in the context of the structural design. Hence, several authors have put forward a simplified modelling approaches for discussion. All models take the stress-strain relationship of SHCC into account, which is significantly different from that of ordinary concrete. The basic assumption of all engineering models for the representation of the material behaviour is the uniform distribution of the properties over the volume considered. Some considerable work exists on the inverse analysis of the experimental investigations of the composite, where the functions used for describing material behaviour range from simple multi-linear approaches [23] to complex mathematical functions [24,25,26]. A formulation based on the work of Kabele [27] has already been included in a commercial structural analysis program.

**Figure 5:**
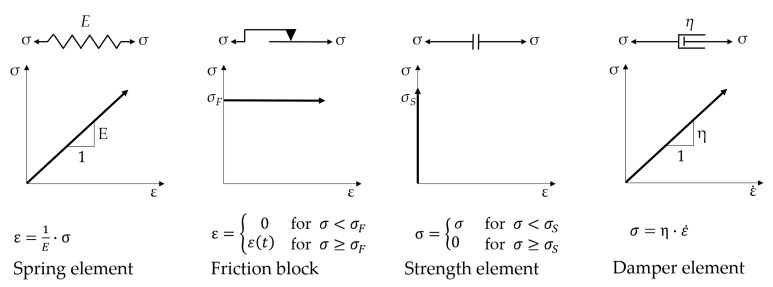
Basic rheological elements and their idealised material behaviours.

A suitable physics-based method for representing the micromechanical behaviour and fracture mechanics of mineral composites is rheological modelling. In this kind of modelling, simple, idealised physical elements such as springs, dampers, strength-elements, or frictional elements are combined to represent material behaviour. These four basic elements are shown in Figure 5 with the respective idealized material behaviour.

Complex material behaviour can be described by a suitable combination of these elements, all of which represent specific features of the material and its damage mechanisms. Such descriptions for the material behaviour of concrete have been suggested by Burgers, and separately Duda [28], among others. A cyclic supplement to existing rheological concrete models has been proposed by Kessler-Kramer [29] and von der Haar [30] for discussion.

The rheological description of single fibre pull-out in considering the fibre properties and the contact zone with bonding bridges according to Butler et al. [31] was suggested by Barhum [32].

## 3. Test Results and Model Interpretation

**Figure 6:**
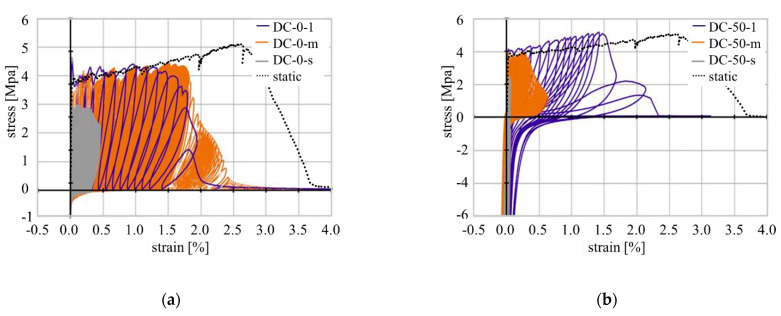
Representative stress-strain curves measured in deformation-controlled cyclic tests compared to a typical quasi-static test: (**a**) pure tension; (**b**) alternating tension-compression with lower reversal point at 50% of the compressive strength.

The macro-mechanical, deformation-controlled and load-controlled cyclic test, by Müller and Mechtcherine [7,14,16], performed under both pure tension and alternating regimes, revealed that SHCC is sensitive to alterations of the experimental settings such as strain increment per cycle or upper and lower reversal points. By increasing the strain increment per loading cycle from 0.00002% to 0.2% the tolerable number of cycles decreased while the total strain increased; see Figure 6a.

Furthermore, a change in the testing regime from pure tension to alternating tension-compression cyclic loading caused a pronounced decrease in strain capacity. While the specimens tested under a strain increment of 0.002% yielded a total strain of 3% in pure tension tests, a compressive component of 50% of the compressive strength evoked a reduction in the strain capacity to approximately 0.6%. With lower strain increments, a much higher number of loading cycles can be sustained by SHCC, thus leading to different degradation processes. These observations hold true for the load-controlled testing regime as well.

**Figure 7:**
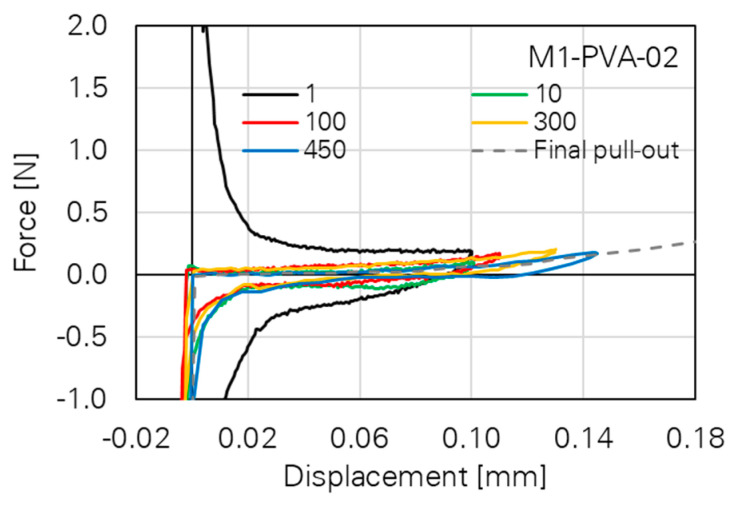
Cyclic, double-sided single fibre pull-out test with PVA fibre [33].

Looking at the single hysteresis loops, a clear change in the inclination angles and, therefore, in the slope of the graph can be seen, indicating a change in material stiffness. Additionally, the loops become wider with increasing strain levels [15]. This was also observed by Ranjbarian and Mechtcherine [33] on the micro-level in fibre pull-out tests; see Figure 7.

Various damage mechanisms could be observed for the SHCC’s constituents, i.e., matrix, fibres, and matrix-fibre-interface, depending on the cyclic loading regime. These mechanisms are explained in the following as a basis for the rheological model of strain-hardening cement-based composites.

### 3.1. Matrix Deterioration

#### 3.1.1. Tensile Matrix Fatigue

With an increasing number of tensile loading cycles, Müller and Mechtcherine [7,16] could observe an increasing level of fatigue in the SHCC matrices regardless of whether the test was deformation-controlled or force-controlled. In the case of sufficiently high upper reversal levels the authors explained this degradation with reference to the gradual growth of micro-cracks in the matrix. Due to the repetitive loading and unloading of the samples, those micro-cracks propagate until they become visible macro-cracks bridged by load-transferring fibres. After this initial crack development in the weakest section of the sample, further cracks can form subsequently in other regions of the sample.

#### 3.1.2. Matrix Spalling

Makita and Brühwiler [34] performed tensile fatigue tests on UHPFRC with more than 10 million cycles. In addition to matrix fatigue, the authors observed matrix spalling as well, especially in the case when the fibre orientation differed from the direction of loading as was described by Li et al. [35] previously.

#### 3.1.3. Microstructural Loosening of the Matrix

**Figure 8:**
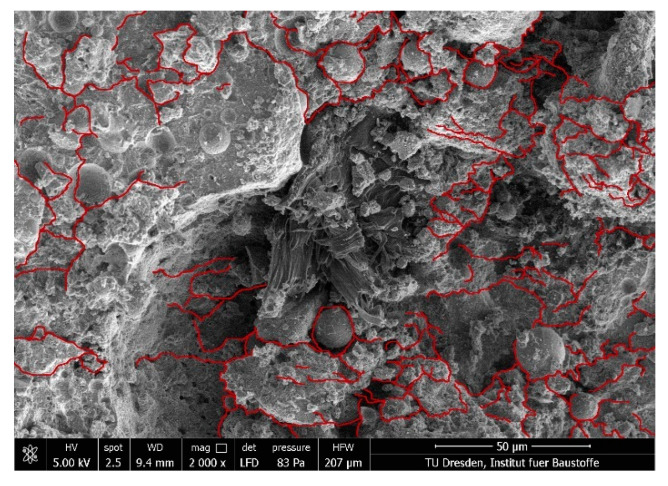
Microcracks in the SHCC matrix after alternating tension-compression loading [7].

The change from a pure tension-swelling regime to alternating tension-compression loading leads to a further damage mechanism in the composite. The investigations by Müller and Mechtcherine [7,14,16] showed that the repeated compression loading causes the development of microcracks and therefore the loosening of the microstructure; see Figure 8.

Due to the loosening of the microstructure, small particles can come free of the matrix and result in further damage to the embedded fibres.

### 3.2. Fibre Degradation

#### 3.2.1. Fibre Fatigue

**Figure 9:**
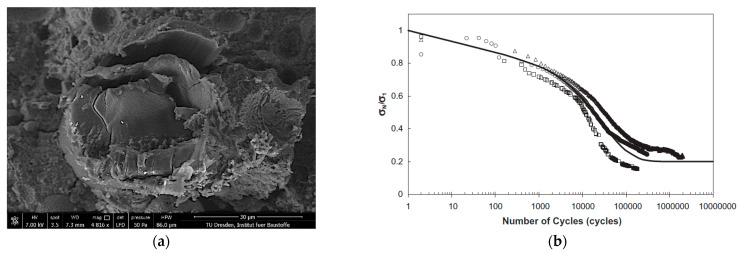
Fibre fatigue: (**a**) fracture surface of a PVA-fibre [7]; (**b**) bridging stress degradation due to fibre fatigue [37].

One possible fibre failure mode is the fibre fatigue that occurs mainly in samples that have sustained a high number of loading cycles. Qiu and Yang [36] conducted fatigue tests on naked PVA fibres and detected a bi-linear load-life-curve with a flat (low-cycle) and a steep (high-cycle) region. Müller and Mechtcherine [7] observed this fibre fatigue failure on specimens in high-cycle, pure tension tests with an upper reversal point of 40% of first-crack strength. In the case of fibre fatigue the surface of the fibres is characterised by striations similar to fatigue fracture surfaces in steel; see Figure 9a. Beside those microscopic investigations, Matsumoto et al. [37] determined an ongoing reduction of the bridging stress due to the increasing fibre fatigue; see Figure 9b.

A high number of loading cycles leads to increasing reduction in a fibre’s cross-section, thus, causing a decrease in its load-bearing capacity.

#### 3.2.2. Defibrillation and Buckling

**Figure 10:**
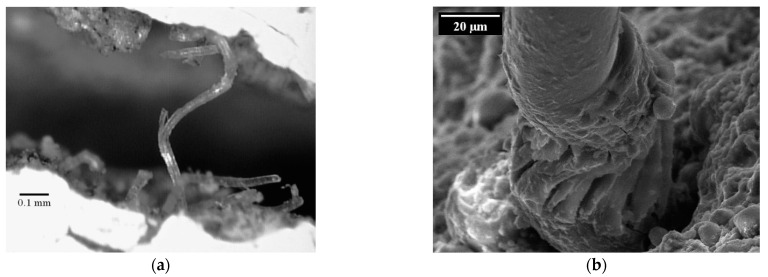
Buckling of crack-bridging fibre in an alternating tension-compression regime: (**a**) microscopic image of a crack [37]; (**b**) a damaged PVA fibre as imaged using electron microscopy [9].

Another degradation mechanism observed by several authors is the defibrillation and buckling of the crack-bridging fibres. Although this phenomenon occurs both in pure tension tests and in alternating tension-compression loading regimes when the cracks are closing in the unloading phase of each cycle, the compressive force causes more pronounced deteriorations of the fibres. Ranjbarian et al. [11] stated a relation between initial crack width and fibre damage due to the forcible closing of the cracks. The authors could observe fibre buckling and eventual defibrillation, i.e., the splicing into individual fibrils, especially at large crack widths, at which the fibres are likely to be fully de-bonded and partially pulled-out in the tension phase. When the cracked specimen is unloaded and then loaded with compressive stresses, the fibres cannot be pushed back into their respective tunnels completely and, thus, are bent or buckled. Due to the lateral expansion of the fibres, longitudinal cracks form [9]. A characteristic example of buckling and defibrillation is shown in Figure 10b. Figure 10a illustrates the significant buckling of the crack-bridging fibre on the macroscopic level, observed by Matsumoto [37].

This degradation mechanism reduces fibre strength significantly [7]. Hence, the number of endurable loading cycles in cracked SHCC decreases with alteration of the loading regime from pure tension cyclic loading to an alternating tension-compression cyclic loading.

The forceful and complete closing of a crack in SHCC induces a further deterioration mechanism in the fibres. In addition to buckling the closure of the crack causes crushing of the fibre between the crack edges [7,14,16] when the fibre is partially extracted from the matrix. This surplus degradation of the fibres results in the decrease in the crack-bridging capacity with increasing number of loading cycles. Thus, an increase in compressive force causes a significant decrease in the number of tolerable loading cycles.

### 3.3. Matrix–Fibre Interface

**Figure 11:**
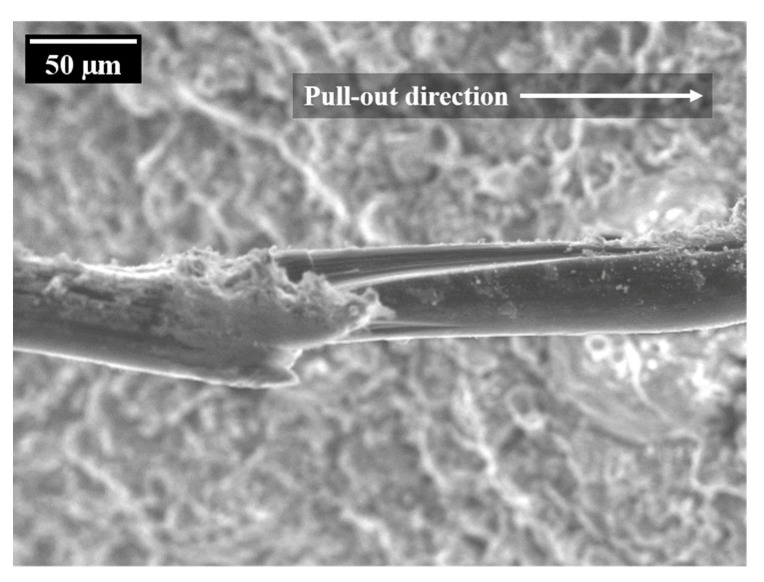
Probable locking front at a PVA-fibre after quasi-static pull-out [40].

Due to the hydrophilic nature of PVA fibres, a relatively strong bond between the fibres and surrounding cement matrix can be observed [38] even if an oiling agent is usually applied to the fibres’ surfaces to minimise bond strength. Furthermore, the interfacial transition zone (ITZ) between fibre and bulk matrix is the weakest part of the composite because of its relatively high porosity. The mechanical properties of the ITZ vary depending on the local conditions in this region. According to Gao et al. [39], there are two failure modes in the ITZ. On the one hand, the fibres can be pulled out due to adhesive failure; this occurs when the shear stress exceeds the shear resistance caused by friction. On the other hand, a cohesive failure type of the ITZ can be observed when the shear resistance is higher than the strength of the transition zone, in this way leading to damage of the ITZ. Ranjbarian and Mechtcherine [40] developed the so-called locking-front model to describe the degradation and pull-out mechanisms of the fibres on the basis of those adhesive and cohesive failure modes. As soon as the de-bonding process is complete, the fibre is pulled out of the matrix. Because of the relatively strong chemical bond between the fibre surface and the cementitious matrix, the authors observed particles adhering to the fibres indicating partial cohesive failure of the interphase. These small matrix particles give rise to transverse pressures on the fibres, thus leading to scratches and finally to micro-excavations. The fibre material from such damage zones accumulates in so-called “bumps” representing the locking front; see Figure 11. Due to the reduction of the fibres’ cross-section and the interlocking of the fibre in the tunnel, the PVA fibres tend to rupture there.

When applying an alternating tension-compression loading regime, damage processes other than fibre rupture can be observed. Ranjbarian and Mechtcherine [9] performed repeated fibre pull-out and push-in movements in a cyclic regime to prevent the development of the locking front. As a result, the fibres tended to be pulled out instead of rupturing.

### 3.4. Modelling of the Deterioration Processes with Rheological Elements

#### 3.4.1. Model Area and Mechanisms

The experimental work has shown a wide range of damage mechanisms for SHCC subjected to cyclic loading. They can be assigned to the main constituents of the composite: fibre, matrix and the interface/interphase between fibre and matrix. A compilation of all relevant mechanisms is given in Table 2.

**Figure 12:**
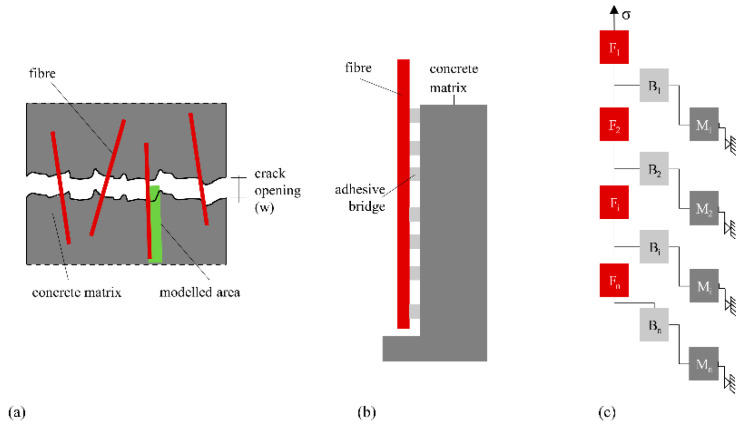
Schematic setup of the modelling approach with (**a**) definition of the modelled region, (**b**) physical model of fibre, matrix and interphase and (**c**) arrangement of idealised entities for rheological modelling.

In this section, a rheological modelling approach is suggested. The basis of this modelling is an individual fibre connected to the concrete matrix via bonding bridges. Both the fibre and the matrix entities as well as the bonding bridges are described by a composition of different basic rheological elements in accordance with [32]. The basic setup of this modelling approach is shown in Figure 12. For modelling the fibre is divided in several sections, 1 to n, while between neighbouring sections an adhesive bridge is placed linking the fibre to the matrix. This bonding bridge can be interpreted as an interphase between fibre and matrix, for example, similar as it forms in the bond zone between aggregate particles and binder matrix. For ordinary concrete such an interphase is usually specified with a thickness of 10 to 50 µm [41]. Investigations of similar cement-based matrices reinforced with PE fibres produced this interphase zone, which, compared to the rest of the matrix, had a significantly looser structure and a thickness of 15 to 30 µm, depending on the type of fibre [42]. In comparison to such an interphase, the concrete matrix appears as a solid, self-contained block. However, this solid block can also be loosened by the cyclic compressive load, resulting in little individual matrix blocks that can only interact with the remaining matrix through frictional forces.

#### 3.4.2. The Concrete Matrix

Von der Haar’s model [30] was chosen as the basis for the concrete matrix’s modelling. The model includes both the linear-elastic part of the deformations and the creep of the material over the loading period. The effects of and damage from cyclic loading and thermal effects were represented by individual elements with defined strain increases, depending on the number of load cycles and the temperature. The thermal element was introduced by von der Haar due to the significant temperature increase measured in high-cyclic compression load regimes. Load regimes considered in the present work with major focus on tensile load component do not develop such a significant temperature increase. In the experiments an increase in temperature of 5 K was measured, which should not affect the mechanical behaviour of the matrix to any significant extent. Nevertheless, the thermal element is still a part of the model since it can become relevant for other loading regimes and material combinations. To represent the physical damage mechanisms better, the black box element is replaced by a formulation very similar to that of creep, which is again composed of both an elastic and a plastic component. This enables implementation of modified damping and stiffness characteristics as a function of the number of load cycles. Additionally, separation of the deformations into plastic and reversible components is possible. Furthermore, loosening of the matrix microstructure was observed, especially in alternating loading tests with high compressive stresses, which led to greater strain of the SHCC before failure. This micro-structural loosening was included in the model using friction elements. They allow for modelling deformation dependent on the number of loading cycles with corresponding energy dissipation. The change in the element depends on the number of load cycles and can be adjusted to the magnitude of the compressive load using a mathematical equation.

**Figure 13:**
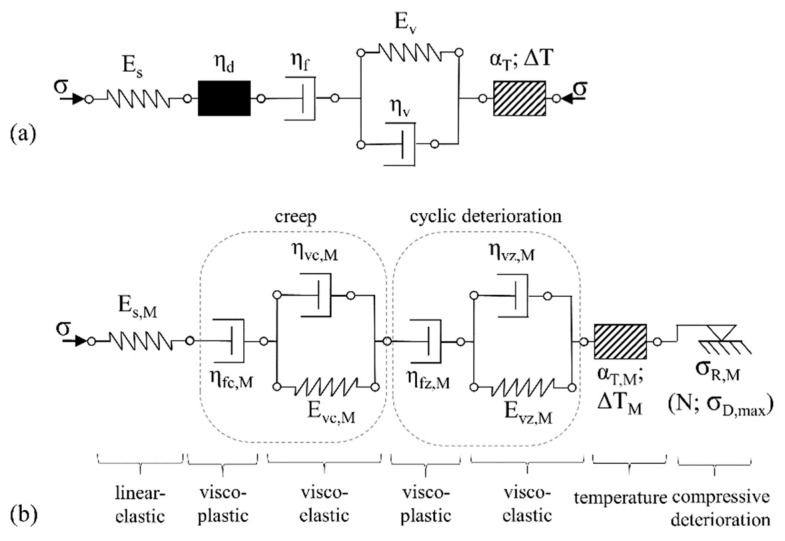
Rheological model for the SHCC matrix (**a**) original model according to [30], (**b**) extended model with detailed representation of cyclic damage.

The resulting scheme of the rheological model for the SHCC matrix is given in Figure 13 and compared to the original model as formulated by von der Haar and Marx [30].

#### 3.4.3. The Interphase

**Figure 14:**
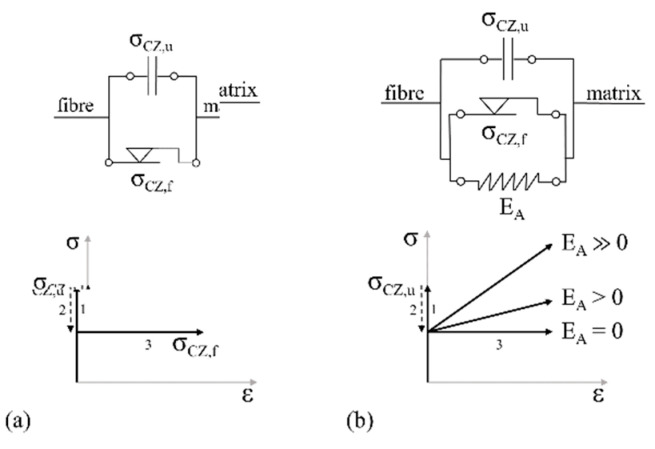
Rheological model of frictional behaviour in the interphase after fibre debonding with idealised stress–strain curves: (**a**) original model according to [32], (**b**) extended model.

The interphase, interface, or contact zone between fibre and matrix was described by Barhum [32] using a serial arrangement of bi-elements/adhesive bridges, each consisting of one frictional element and one strength element connected in parallel. The strength element stands for the maximum stress transfer by the undamaged interphase while the friction element represents a stress transferred due to friction after the adhesive bridge has broken. Usually, the frictional stress is clearly below the shear strength of the interphase. Microscopic examinations of the fracture surfaces of SHCC tested in both cyclic and quasi-static regimes show clear signs of abrasion on the fibre surface. The rubbed-off material remains in the fibre channel and narrows it. The result is an increasing friction component in the space of the constriction, which cannot be described by the existing elements. Thus, the frictional component of the interphase is extended in the modified model by a spring element connected in parallel. Depending on the stiffness of the spring element, the increase in force of the friction component can remain constant (E = 0) or increase (E > 0) with increasing pull-out displacement. This extension allows for the inclusion of effects related to fibre erosion; see the Locking front model [41]. By assigning a proper statistical distribution/probability function to all involved adhesive bridges, all experimentally observed pull-out phenomena can be described by the model in respect of abrasion, the associated friction, and so-called slip-hardening [43] effects. The graphical adaptation of the element group and idealised stress-strain (force-elongation) curves depending on the spring stiffness are shown in Figure 14.

According to Boshoff and van Zijl [44,45], a large portion of the creep deformations of cracked SHCC is due to the enlargement of existing cracks, which affects the contact zone between fibre and matrix. Therefore, the model for the application in SHCC should be extended by a creep component, which in turn consists of a reversible creep component and an irreversible one. The cause of the creep deformation observed by Boshoff and van Zijl is not yet clearly understood. They quantified the creep of the fibre material itself as having little relevance. Thus, on the one hand, creep deformation of the interphase is possible. On the other hand, the coating material of the PVA-fibre may also contribute to fibre displacement and crack opening. Finally, there is a possibility that the creep deformations as recorded are due to progressive fibre delamination and (partial) fibre pull-out. On the model side, an element group for creep was inserted with the parameter set of the SHCC matrix in addition to linear-elastic behaviour. Obviously, the properties of the interphase are likely to depart from those of the matrix. Thus, some adjustment might be needed with respect to this parameter set.

An additional extension of the model was possible by introducing a cyclic damage component. Both the interphase region and the concrete matrix have the same material basis and analogous physical damage processes. An analogous representation of the cyclical damage processes by rheological elements is therefore appropriate. In comparison to the matrix, the interphase exhibits higher porosity and lower content of aggregate particles in its composition. Hence, the coefficients and variation parameters must be adjusted accordingly.

Additionally, the interaction between fibre and matrix does not depend on the properties of the interphase only. The sizing or coating of the fibre material plays an important role, too. Normally some polymer-based or other organic formulations are used, whose properties are pronouncedly temperature dependent. To separate the described effects, the interface model was separated into one part representing the cementitious interphase and another part representing the contact interface, which also includes the effects of interlocking.

**Figure 15:**
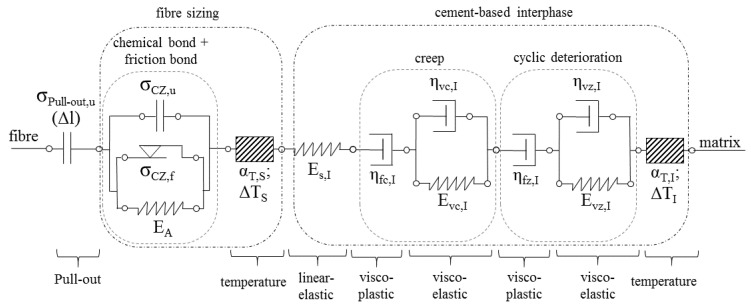
Rheological model of the interphase/contact zone with all introduced extensions.

Finally, fibre pull-out can lead to the loss of contact between the fibre and the adhesive bridge. This effect is represented in the model by a strength element which loses its strength when a particular pull-out displacement is reached. This means that the element transmits all applied stresses until the fibre pull-out has progressed so far that there is no longer any geometric contact between the fibre and the bonding bridge element remaining in the fibre channel. The complete scheme of the model for the contact zone is shown in Figure 15.

#### 3.4.4. The Fibre

**Figure 16:**
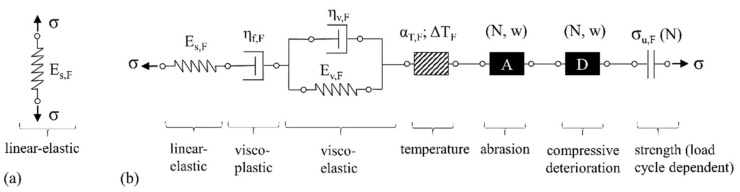
Rheological models of the fibre: (**a**) original model according to [32], (**b**) suggested extension including known damage mechanisms.

Barhum [32] used a spring element to describe the fibre properties as linear-elastic; see Figure 16a. Clearly the damage mechanisms of the individual components as listed in Table 2 cannot be differentiated using this approach. Thus, all known phenomena must be addressed independently in the model. For example, the mechanical properties of the PVA fibre strongly depend on temperature [43,46]. Another effect to be taken into account is the observed abrasion of fibre material during pull-out, as already described in Section 3.4.3. This degradation mechanism is not only associated with a clamping effect on the fibre channel wall, but also, on the fibre side, with a reduced residual cross-section of the fibre due to the notch and a stress concentration at the notch. The extent of this phenomenon depends significantly on pull-out displacement, whereby the additional effects of cyclic loading cannot be excluded given the current state of knowledge. In addition to abrasion, pronounced destruction of the fibre material was observed during alternating cyclic loading due to the forceful closing of the crack flanks and associated crushing of the firstly pulled-out and then compacted fibre material. This degradation is only localised at the crack plane and directly depends on the number of load cycles and the magnitude of the compressive load. Finally, cyclic damage with a reversible and an irreversible component can also be expected for polymer fibres.

By assuming that these damage types follow the superposition principle, i.e., a summation of the strain components, the phenomena described were added to the model by a serial connection; see Figure 16b.

**Figure 17:**
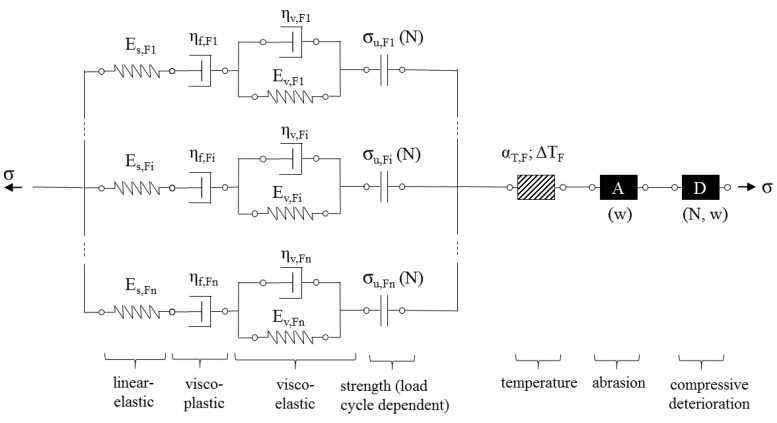
Rheological model of the fibre including the damage mechanism defibrillation.

In addition to the fibre-damage mechanisms considered above, a special form of fatigue damage was observed when investigating the fracture surfaces of fibres. Fibres subjected to particularly high numbers of load cycles tend to defibrillate, i.e., the closed fibre strand separates into many smaller parallel entities, so-called fibrils, as can be seen, for example, in Figure 10b; see also [41]. Such a splicing of the compact fibre cross-section leads to a reduction in both the fibre’s stiffness and its load-bearing capacity since only a part of the fibrils/cross-section is (fully) involved in the force transfer. Variations in the tensile forces carried by individual fibrils and in their properties play a prominent role in conjunction with missing stress transfer points between the fibrils. To describe this damage mechanism within the extended model, the fibre should be considered as a sum of parallel fibrils (1 to n), to which varying mechanical parameters are assigned. The influencing variables from abrasion, pressure damage, and temperature influence, which cannot be described more precisely at this stage, are added to the fibril elements as series elements. The resulting model is shown in Figure 17.

#### 3.4.5. The Complete Model

**Figure 18:**
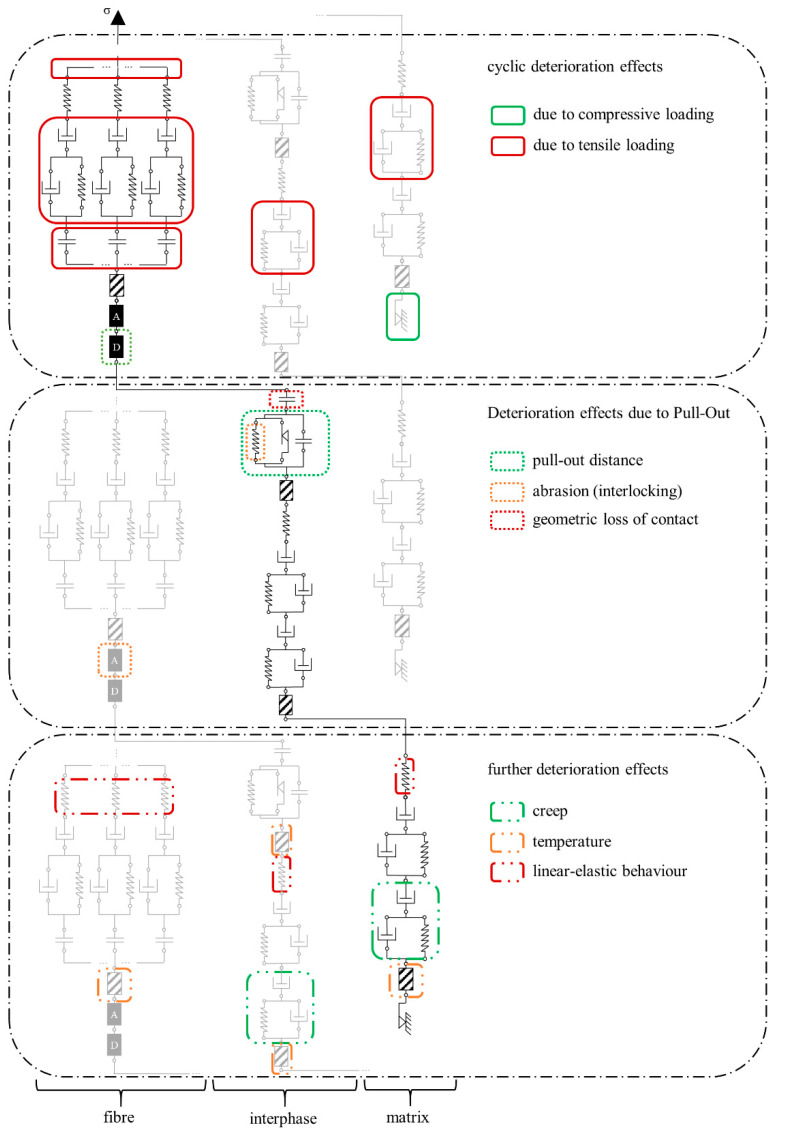
Complete rheological model with highlighted effects on the strain, one load path is highlighted in black, the incomplete displayed load paths are light grey. Each element group occurs several times (three times are displayed here).

All model adjustments suggested are shown in Figure 18. The respective final arrangement of element groups is analogous to the basic idea presented in Figure 12, a row of element groups for fibre, interphase and matrix with specific connections between the groups. The fibre is divided into several sections. Between two neighbouring fibre sections is positioned one interphase entity, which is always connected to a matrix element. For the sake of readability, only three sections of a fibre as well as the corresponding interphase/contact zone and matrix sections are shown. Furthermore, one force path is highlighted. In addition, various exposures are displayed, and the corresponding reaction and possible damage location are highlighted.

The present model is relatively complex, so that its mathematical description cannot be included in this publication due to the inordinate number of formulas and the numerous assumptions to be presented. While not all parameters needed for subsequent calculation are known at this stage, it seems still to be helpful to apply the model using sensibly made assumptions. The calculation results generated in this way for the fibre pull-out under cyclic loading offer the possibility of estimating the effect of individual parameters’ changes on the overall response of the system. This work is to be presented in a follow-up publication. The results of the presented physically based rheological model can be transferred to the material’s representative volume such as tensile test specimen by means of modelling approaches similar to those suggested by Barhum [32] or Jun and Mechtcherine [17] when sufficient test results are available.

## 4. Discussion

Even without being able to present mathematical calculations in this publication, it can be stated that the pull-out model as developed offers the advantage of assigning different failure mechanisms to a specific region of effect, i.e., fibre, interphase/contact zone or matrix. This improves the understanding and the description of the material behaviour and, subsequently, provides possible starting points for material optimisation as well.

The observation of the failure ranges of the cyclically loaded specimens showed similar behaviour at low numbers of loading cycles in comparison to quasi-statically loaded specimens with the main failure mechanism of fibre pull-out. This case was described as Mode I. If the number of load cycles increases and an additional pressure component is introduced into the cycle, degradation can occur in the partially extracted fibres due to crushing processes at the crack flanks, which was defined as Mode II. If, in addition, the compressive forces at the lower reversal point are set higher, the fibres degrade, while the matrix is damaged, and the microstructure loosens in the crack area. This failure mechanism is considered as Mode III. Fracture surface investigations of specimens subject to high numbers of loading cycles show, in addition to the failure modes already described, fibre fatigue as Mode IV failure, which is characterised by fibre failure with typical fatigue fracture patterns and fibre defibrillations. In summary, on the basis of the cyclic tests’ results and the determined degradation mechanisms, the following four different failure modes can be identified, depending on the number of load cycles and load levels:

- Mode I: fibre pull-out;
- Mode II: fibre degradation;
- Mode III: fibre degradation accompanied by matrix damage;
- Mode IV: fibre fatigue.

In accordance with the Mode I mechanism found, i.e., fibre pull-out, the failure is mainly defined by the interphase/contact zone. However, the abrasion of fibre material can affect the fibre through the reduction of its cross-section and stress peaks.

In contrast, for the Mode II mechanism, fibre degradation, the fibre properties are the determining factor. They include the stiffness and tensile strength of the fibre, which in turn depend on the regime of cyclic loading.

The Mode III mechanism is based on the same damage process as in Mode II, except that additional matrix properties are altered by the introduction of compression components which lead to loosening of the matrix. Furthermore, the destruction of the fibre by compressive loading in the crack plane plays an essential role in this damage mechanism.

For fibre fatigue in Mode IV, the fibre properties become absolutely decisive.

Other important parameters that contribute to the observed deformations are creep, linear-elastic strains, and the influence of temperature. However, they can only be quantified by means of further extensive experimental work and cannot, therefore, be evaluated independently in the present work.

The damage mechanisms described in this article are obviously more numerous than those considered in previous modelling approaches. Such a comprehensive description of all relevant mechanisms and parameters is certainly very complex and bulky. However, it is certainly needed to cover comprehensively the effects of various system parameters such as material composition, loading regime, etc. on the mechanical performance of the composite. The model as presently developed includes all experimental mechanisms which have been discovered and understood. In the following step, the model will be transformed into a set of mathematical formulas and used for calculations under implementation/assumption of adequate input parameters.

## 5. Conclusions

The article at hand has presented a new rheological model for strain-hardening, cement-based composites (SHCC) subjected to alternating tension-compression loading. To develop the model, the authors analysed experiments performed on SHCC on different scales to define the major types of deterioration mechanism in the material. Subsequently, from these mechanisms a rheological model was derived to reflect the micro-mechanics of SHCC. Since specific behaviour and deterioration phenomena were found for three main phases in the material, i.e., fibre, fibre-matrix interphase and cement-based matrix, the model consists of three distinct parts corresponding to these phases. It also considers a variety of deterioration triggers for each of the abovementioned phases, e.g., loading type, fibre pull-out, creep, and temperature. Due to a clear distinguishing of phases and degradation mechanisms, the material can be purposefully optimised using the comprehensive model.

Still further, the model depicts knowledge deficits for a quantitative description of material behaviour based on real failure mechanisms. While complete data sets for all mechanisms have yet to be identified, the model framework already enables some qualitative analysis suitable for improving the material resistance to cyclic loading.

In this context, the fibre properties and their bonding properties with the concrete matrix represent significant adjustment options. For example, a complete closing of the crack can be effectively prevented by using stiffer fibres and thus the failure mechanism related to crushing the fibres between crack edges can be hindered, or at least diminished. Furthermore, improved fibre integration into the concrete matrix, for example, by fibre-end anchors, seems to present an effective method to increase fatigue strength. To improve the existing database, tests on the creep of the individual fibres, the interphase and the matrix material should be carried out in near future. In addition, the influences of strain rate, temperature, and fibre orientation have not yet been conclusively clarified.

## Figures and Tables

**Table 1:** Composition of the SHCC under investigation, in kg/m³.

Cement CEM I 42.5 R-HS	Fly Ash	Water	Quartz Sand 0.06/0.2	Superplasticizer	Viscosity Agent	PVA Fibre 2.0 vol.%
505	621	338	536	8.5	3.2	26

**Table 2:** Experimentally specified deterioration mechanisms.

Fibre	Cement-Based Matrix	Interphase
Tensile rupture	Tensile rupture	Tensile rupture
Fibre fatigue	Compression failure	Fatigue
Defibrillation	Fatigue	Creep
Creep	Creep	Thermal effects
Abrasion	Thermal effects	-
Grinding	-	-
Thermal effects	-	-
